# Three-Dimensional Bioprinted Gelatin—Genipin Hydrogels Enriched with hUCMSC-Derived Small Extracellular Vesicles for Regenerative Wound Dressings

**DOI:** 10.3390/polym17091163

**Published:** 2025-04-24

**Authors:** Manal Hussein Taghdi, Maimonah Eissa Al-Masawa, Barathan Muttiah, Mh Busra Fauzi, Jia Xian Law, Ani Amelia Zainuddin, Yogeswaran Lokanathan

**Affiliations:** 1 Department of Tissue Engineering and Regenerative Medicine, Faculty of Medicine, Universiti Kebangsaan Malaysia, Cheras, Kuala Lumpur 56000, Malaysia; manalhussein240@gmail.com (M.H.T.); maimonah.almasawa@gmail.com (M.E.A.-M.); barathanmuttiah@ukm.edu.my (B.M.); fauzibusra@ukm.edu.my (M.B.F.); lawjx@hctm.ukm.edu.my (J.X.L.); 2 Department of Anesthesia and Intensive Care, Faculty of Medical Technology, University of Tripoli, Tripoli P.O. Box 13932, Libya; 3 Advance Bioactive Materials-Cells UKM Research Group, Universiti Kebangsaan Malaysia, Bangi 43600, Malaysia; 4 Department of Obstetrics and Gynecology, Faculty of Medicine, Universiti Kebangsaan Malaysia, Cheras, Kuala Lumpur 56000, Malaysia; aniamelia@hctm.ukm.edu.my

**Keywords:** extracellular vesicles, gelatin, 3D bioprinting, 3D biomaterial printing, biomaterials ink, skin regeneration, cutaneous regeneration

## Abstract

Mesenchymal stromal cell-derived small extracellular vesicles (MSC-sEVs) have shown great promise in promoting tissue repair, including skin wound healing, but challenges like rapid degradation and short retention have limited their clinical application. Hydrogels have emerged as effective carriers for sustained EV release. Three-dimensional printing enables the development of personalized skin substitutes tailored to the wound size and shape. This study aimed to develop 3D bioprinted gelatin–genipin hydrogels incorporating human umbilical cord MSC-sEVs (hUCMSC-sEVs) for future skin wound healing applications. Gelatin hydrogels (8% and 10% *w*/*v*) were crosslinked with 0.3% genipin (GECL) to improve stability. The hydrogels were evaluated for their suitability for extrusion-based 3D bioprinting and physicochemical properties, such as the swelling ratio, hydrophilicity, enzymatic degradation, and water vapor transmission rate (WVTR). Chemical characterization was performed using EDX, XRD, and FTIR. The hUCMSC-sEVs were isolated via centrifugation and tangential flow filtration (TFF) and characterized. The crosslinked hydrogels were successfully 3D bioprinted and demonstrated superior properties, including high hydrophilicity, a swelling ratio of ~500%, slower degradation, and optimal WVTR. hUCMSC-sEVs, ranging from 50 to 200 nm, were positive for surface and cytosolic markers. Adding 75 μg/mL of hUCMSC-EVs into 10% GECL hydrogels significantly improved the biocompatibility. These hydrogels offer ideal properties for 3D bioprinting and wound healing, demonstrating their potential as biomaterial scaffolds for skin tissue regeneration applications.

## 1. Introduction

Wound healing is a complex process involving coordinated signaling and interactions between various cell types and the extracellular matrix to restore tissue structure and function. Full-thickness wounds are particularly challenging to repair and pose serious health risks [1]. The increasing prevalence of non-healing wounds, driven by obesity, diabetes, and aging, places a significant burden on healthcare systems and the economy [2,3]. Traditional wound dressings are often inadequate for managing complex wounds, highlighting the need for advanced regenerative approaches. Mesenchymal stem cells (MSCs) have been widely explored for skin repair because of their regenerative properties and ability to enhance wound healing [4]. MSCs can be derived from various sources, including bone marrow, adipose tissue, and umbilical cords. Among these, human umbilical cord MSCs (hUCMSCs) stand out due to their easy procurement, high proliferative capacity, and lack of ethical concerns [5]. However, challenges such as tumor formation risks, immune rejection, and long-term clinical complexities hinder MSC-based therapies [4]. Emerging research suggests that mesenchymal stem cells (MSCs) primarily exert their therapeutic effects through paracrine signaling, with extracellular vesicles (MSC-EVs) playing a critical role in mediating cell communication and signaling. These vesicles are key carriers of bioactive molecules that facilitate intercellular communication, influencing various biological processes such as tissue regeneration, immune modulation, and cellular repair mechanisms [6]. MSC-EVs contain bioactive molecules, including mRNA, miRNA, proteins, and lipids, which regulate cellular processes and facilitate tissue repair [7,8,9]. hUCMSC-derived small extracellular vesicles (hUCMSC-sEVs) have demonstrated potential in stimulating all phases of wound healing, including hemostasis, inflammation, proliferation, and remodeling [10,11]. However, their therapeutic efficacy alone may be insufficient for large or full-thickness wounds, which require both biochemical signaling and structural support [12]. Chronic conditions such as aging and diabetes further complicate healing by impairing cellular responses [13,14]. Biomaterials such as hydrogels provide a three-dimensional structure that supports cell attachment, migration, and tissue formation. Hydrogels help maintain a favorable wound environment by retaining moisture and absorbing excess exudate while gradually degrading to facilitate tissue integration [15]. This approach aligns with the principles of tissue engineering, which combine biomaterials, cells, and bioactive molecules to promote functional tissue regeneration [16,17]. Hydrogels also enable the controlled, localized release of MSC-EVs, preventing their rapid clearance and enhancing their regenerative potential [12,18]. This combination addresses both the structural and biochemical requirements for effective wound healing, particularly in chronic and large wounds. Gelatin hydrogels, in particular, have shown promise in combination with MSC-EVs [10,18,19]. As a collagen-derived biopolymer, gelatin is widely used in wound care because of its natural degradability, minimal immune response, and resorbability [20,21,22]. Additionally, it is cost-effective and can be modified to enhance its properties. However, its weak mechanical strength limits its application [23]. Crosslinking is commonly employed to reinforce gelatin hydrogels, improving their thermal and mechanical stability [24]. Although chemical crosslinkers like glutaraldehyde are effective, they pose cytotoxicity risks due to potential leaching during degradation [25]. As a safer alternative, genipin (GNP), a naturally occurring crosslinker derived from Gardenia jasminoides, has gained attention for its ability to enhance gelatin’s properties without toxic effects [26,27]. This shift toward natural crosslinkers aligns with the growing interest in sustainable and biocompatible biomaterials for wound healing [28]. Beyond biomaterials and MSC-EVs, advanced 3D printing technologies further enhance wound healing strategies. Three-dimensional bioprinting enables the fabrication of highly customized hydrogel scaffolds tailored to the wound size, shape, and specific patient needs [29]. Techniques such as extrusion-based bioprinting, inkjet bioprinting, and stereolithography offer precise scaffold structuring, supporting cell encapsulation and tissue regeneration [30]. Additionally, imaging techniques like computed tomography (CT) provide data for precise digital wound modeling, optimizing hydrogel application, and improving overall healing outcomes [31]. By integrating 3D bioprinting with hydrogel-based MSC-EV delivery, researchers can develop highly efficient, patient-specific solutions for treating complex and chronic wounds. This research takes an integrated approach by combining advanced 3D bioprinting with a biocompatible gelatin–GNP hydrogel infused with hUCMSC-sEVs, aiming to fabricate precisely designed scaffolds that provide both structural support and a regenerative microenvironment for skin repair. Specifically, this study will investigate the physicochemical properties of the gelatin–GNP hydrogel and the biocompatibility of the gelatin–GNP hUCMSC-sEVs combination for potential future clinical applications. By addressing both the structural and biochemical needs of wound healing, this strategy offers an effective solution for treating large and chronic wounds.

## 2. Materials and Methods

### 2.1. Ethical Approval

**Figure 1:**
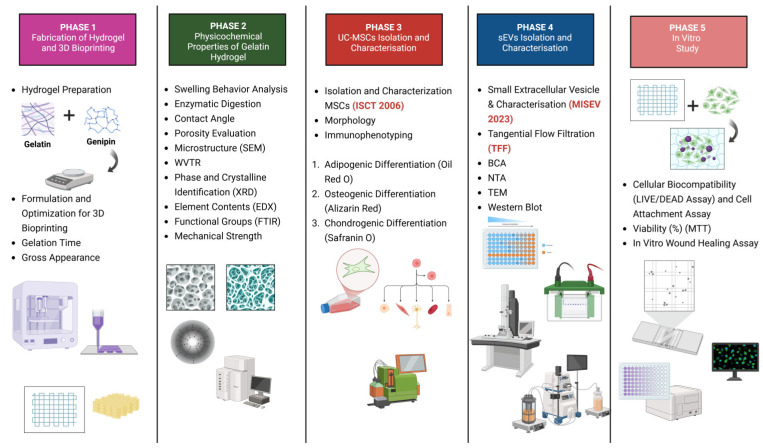
This study follows a structured five-phase approach to develop and evaluate human umbilical cord stem cell-derived small extracellular vesicle (hUCSMC-sEV)-incorporated gelatin hydrogels for cellular biocompatibility assessment.

This study received ethics approval from the Research Ethics Committee Universiti Kebangsaan Malaysia (approval code no.: JEP-2023-421). Written informed consent was obtained from all donors. All experimental procedures adhered to the relevant guidelines and regulations. Figure 1 is a schematic illustration of the experimental design of this study.

### 2.2. Materials

Commercial-grade gelatin (GE) powder was procured from Nitta Gelatin Ltd. (Yao City, Osaka, Japan) and used as the primary biomaterial for the fabrication of the gelatin-based hydrogel. The natural crosslinking agent genipin was purchased from FUJIFILM Wako Pure Chemical Corporation (Chuo-ku, Osaka, Japan) and prepared in 70% ethanol (MERCK, Darmstadt, Germany) for hydrogel crosslinking. For the swelling ratio evaluation, phosphate-buffered saline (PBS) powder was obtained from Sigma-Aldrich (St. Louis, MO, USA) and reconstituted as a buffer medium. To assess enzymatic degradation, a 0.0006% (*w*/*v*) collagenase type I solution (Worthington Biochemical Corporation, Lakewood, NJ, USA) was used to simulate the in vitro enzymatic conditions. Distilled water was used as the solvent in various physicochemical characterization tests. Primary human umbilical cord mesenchymal stem cells (hUCMSCs) and their derived small extracellular vesicles (hUCMSC-sEVs) were used for in vitro biological assays. Primary human dermal fibroblasts (PHDFs) were used to evaluate the biocompatibility of the hydrogel systems. Cell viability was examined via qualitative live/dead staining using the LIVE/DEAD™ Viability/Cytotoxicity Kit (Invitrogen, Waltham, MA, USA), while quantitative analysis was performed using the MTT assay (Sigma-Aldrich, St. Louis, MO, USA) to determine the cell metabolic activity.

### 2.3. Hydrogel Preparation and 3D Bioprinting

To prepare the gelatin solution, different concentrations (8% and 10% *w*/*v*) were prepared by dissolving the gelatin powder (GE; Nitta-Gelatin Ltd., Yao City, Osaka Japan) in distilled water by continuous stirring at 37 °C using a magnetic stirrer set to 400 rpm for 30 min. After dissolution, the gelatin solution was incubated at 37 °C for an additional 30 min to ensure complete homogeneity. Meanwhile, a 0.3% (*w*/*v*) genipin (GNP) solution was prepared by dissolving GNP (Fujifilm Wako Pure Chemical, Chuo-ku, Osaka, Japan) in 10 mL of 70% ethanol (EtOH; Merck, Darmstadt, Germany). Polymerization times were assessed using the inverted tube test at 22–24 °C.

The hydrogel biomaterial ink was prepared in four groups based on the GE concentration and crosslinking state. To create the crosslinked GE–GNP hydrogel biomaterial ink, the GE and GNP solutions were mixed in a 3:1 ratio at 37 °C, resulting in a crosslinked solution (GECL). There were two crosslinked hydrogel bioink groups, 8% GECL (8% GE + 0.3% GNP) and 10% GECL (10% GE + 0.3% GNP), and two non-crosslinked bioink hydrogel (GNC) groups, 8% GNC (GE 8%, *w*/*v*) and 10% GNC (10%, *w*/*v*). The prepared hydrogel was loaded into a 10 mL syringe fitted with a nozzle of 400 μm diameter. The extrusion 3D bioprinting system Biogens XI (3D Gens, Shah Alam, Selangor, Malaysia) was used for the 3D bioprinting process. Autodesk Fusion AutoCAD version 9.20.0.0software (stl file format) was used to create the model. The designed structures were put into Simplify3D software (version 4.1). To maintain the biomaterial ink in a sol-gel state, the printing head temperature was controlled at 25 °C, allowing the ink to remain printable for the required duration. A CAD (Autodesk Fusion AutoCAD 2021 software) design was employed to generate a net structure with dimensions of L10.0 × W10.0 × H0.25 mm^3^ per layer. Using this design, a cubic scaffold consisting of 4 layers was fabricated. Before initiating the bioprinting process, the printing feed rate and pattern parameters were optimized to ensure uniform extrusion and a precise scaffold architecture.

### 2.4. Visual Assessment of the Gross Morphology

The 3D bioprinted hydrogels were visually assessed immediately after the polymerization process to evaluate their external morphology and uniformity. Using a digital camera, detailed images were captured to document the differences between the crosslinked and non-crosslinked samples.

### 2.5. Hydrogel Physical and Chemical Properties Assessment

#### 2.5.1. Swelling Behavior

The swelling behavior of the hydrogels was investigated by submerging them in PBS at 37 °C for 6 h. Swelling ratios were calculated based on the weight difference before and after immersion, providing data on the hydrogel’s capacity to absorb fluids. The swelling ratio (SR) was calculated experimentally using Formula (1):

(1) $$Swelling\;Ratio\;(\%)(\%)=\frac{W^{2}-W^{1}}{W^{1}}\times 100$$

where W^2^ is the swollen weight of the hydrogels, and W^1^ is the dry weight of the hydrogels. The swelling ratio is the fractional increase in the weight of the hydrogel caused by water absorption.

#### 2.5.2. Porosity Evaluation

The porosity of the freeze-dried hydrogels was evaluated using a combination of scanning electron microscopy (SEM) and the liquid displacement method.

SEM, operated at 15 kV, was employed to examine the surface morphology and internal structure of the hydrogels. SEM was used for the high-magnification imaging, providing detailed insights into the fibrous and microporous architecture.

In addition, the liquid displacement method was used to quantify the porosity. Ethanol served as the displacement medium, allowing for the measurement of the porosity percentage.

The lyophilized hydrogels were first weighed (M1) before being immersed in 99.5% ethanol for 24 h. Following immersion, excess ethanol was carefully removed using Whatman^®^ No. 42 filter paper (Merck, Darmstadt, Germany), and the weight of the hydrogel (M2) was then recorded.

The porosity was calculated using Formula (2):

(2) $$Porosity\;(\%)=\frac{M2-M1}{pV}\times 100$$

where p is the density of 99.5% EtOH, V is the volume of the hydrogel, and M1 and M2 are the initial and final weights of the lyophilized hydrogels, respectively.

#### 2.5.3. Water Vapor Transmission Rate (WVTR)

This study was conducted on the basis of the American Society for Testing and Materials (ASTM) standard [32,33]. In brief, the hydrogels were placed on the opening of a glass vial that contained 10 mL of dH_2_O. The samples were placed in a controlled environment (5% CO_2_ at 37 °C). The water vapor transmission rate was recorded and calculated as shown in Formula (3) below:

(3) $$WVTR(g/m^{2}\cdot hour)=\frac{Wi-Wf}{A\cdot time}$$

where *Wi* is the initial weight, *Wf* is the final weight, and *A* is the surface area of the glass vial.

#### 2.5.4. Contact Angle and Wettability Analysis

The contact angle was measured to determine the surface wettability with distilled water droplets by dropping 10 µL of dH_2_O onto the surface of the hydrogel, and the angle was analyzed. The contact angle analysis performed using the ImageJ application (V1.5, Bethesda, MD, USA) revealed the hydrophilic or hydrophobic nature of the samples, which influences cell attachment and proliferation.

#### 2.5.5. Enzymatic Biodegradation

The biodegradability of the hydrogels was analyzed by immersing the samples in 0.0006 mg/mL collagenase type I (Worthington-Biochemical Corporation, Lakewood, NJ, USA) in Dulbecco’s phosphate-buffered saline (DPBS) solution (Gibco, San Diego, CA, USA) at physiological temperature (37 °C) for 24 h. The weight loss of the hydrogels over time served as an indicator of enzymatic breakdown, simulating the in vivo microenvironment. The samples were rinsed, dried, and reweighed to calculate the percentage weight loss. This method provided insight into the hydrogel’s degradation profile, which is critical for determining its suitability as a biomaterial for regenerative applications. The percentage of weight loss was calculated as shown in Formula (4):

(4) $$Weigth\;Loss\;\left(\%\right)=\frac{W^{1}-W^{2}}{W^{1}}\times 100$$

where W^1^ is the initial weight, and W^2^ is the final weight.

### 2.6. Chemical Analysis

#### 2.6.1. Energy-Dispersive X-Ray

The elemental composition of the hydrogel surface was analyzed using Energy-Dispersive X-ray (EDX) using a Phenom Pro X SEM EDX microscope (Phenom, Eindhoven, The Netherlands).

#### 2.6.2. Fourier Transform Infrared Spectrophotometry

Fourier Transform Infrared (FTIR) spectroscopy was employed to identify the functional groups within the hydrogels, confirming the chemical modifications introduced by crosslinking. Measurements were conducted using an FTIR spectrophotometer (PerkinElmer, Waltham, MA, USA) in the range of 4000–500 cm⁻^1^, with a resolution of 2 cm⁻^1^ per point at room temperature.

#### 2.6.3. X-Ray Diffraction Study

X-ray diffraction (XRD) analysis of the sample was conducted at room temperature using an advanced X-ray diffractometer (Bruker AXS GmbH, Karlsruhe, Germany) in θ–2θ scan mode. The diffraction patterns were recorded with CuKα radiation (λ = 1.542 Å) under operating conditions of 35 kV and 10 mA. The sample was scanned over a 2θ range from 10° to 70° in a constant scan mode. The diffractogram was further analyzed using the integrated software (Diffrac. Suite EVA, V4.0, Bruker, Coventry, UK) to identify specific peaks and interpret the results.

#### 2.6.4. Mechanical Testing (Compression Test)

To assess the mechanical properties of the hydrogel biomaterials, specifically their elastic limit and compressive strength, a compression test was performed. This method involves placing each hydrogel sample on a flat surface and applying a defined load to evaluate the material’s response to stress. The test was designed to examine the behavior of the biomaterials under both constant and progressively applied loads, measuring the maximum stress they can withstand over time and providing insights into their resilience and structural integrity under mechanical forces.

The hydrogel samples, fabricated according to the optimized formulations, were polymerized and maintained at room temperature before testing. The mechanical properties were evaluated using a high-precision universal testing machine (Autograph AG-X, Shimadzu, Japan) at a crosshead speed of 5 mm/min. Cylindrical samples (15 mm in diameter, 10 mm in height) were employed to ensure the consistency and reproducibility of the measurements. The compressive strength was determined by calculating the stress values at the maximum load, offering a comprehensive understanding of the material’s mechanical behavior and suitability for load-bearing applications, such as tissue repair and implantable biomaterials.

### 2.7. Isolation and Culture of hUCMSCs

The umbilical cords (n = 3) were collected from healthy full-term births, of mothers aged 25–40 years at the Hospital Canselor Tuanku Muhriz, Universiti Kebangsaan Malaysia, Malaysia, during natural birth and elective cesarean sections. Mothers with conditions such as gestational diabetes or hypertension were excluded. Umbilical cords (6–10 cm) were disinfected, and vascular structures were removed before mincing the tissue into ~2 mm^2^ pieces. The tissue was washed with DPBS (Gibco, Carlsbad, CA, USA), and digestion was performed using 0.6% collagenase type I (Worthington-Biochemical Corporation, Lakewood, NJ, USA) at 37 °C with shaking for 1 h. Cells were cultured in α-MEM (Gibco, Weilam Rhein, Germany) supplemented with 10% in-house human platelet lysate and 1% antibiotic-antimycotic (Gibco, Weilam Rhein, Germany), with media changes every 3 days until 70–80% confluency. Expanded cells were dissociated at passage 2 with 0.05% trypsin-EDTA (Gibco/BRL, Carlsbad, CA, USA). hUCMSCs were characterized in accordance with the criteria established by the International Society for Cell and Gene Therapy [34]. Immunophenotyping of hUCMSCs was performed using flow cytometry (BD FACSVerse™, Franklin Lakes, NJ, USA) and the Human MSC Analysis Kit (BD Biosciences, Franklin Lakes, NJ, USA) to check for the presence of positive MSC markers (CD44, CD73, CD90, and CD105) and absence of negative markers (CD11b, CD19, CD34, CD45, and HLA-DR). Trilineage differentiation was induced using the StemPro™ (Osteogenesis/Chondrogenesis/Adipogenesis) Differentiation Kit (Gibco, Weilam Rhein, Germany) following the manufacturer’s protocol and confirmed by staining: Alizarin Red (Sigma-Aldrich, St. Louis, MO, USA) for osteogenesis, Oil Red O (Sigma-Aldrich, St. Louis, MO, USA) for adipogenesis, and Safranin O (Sigma-Aldrich, St. Louis, MO, USA) for chondrogenesis.

### 2.8. hUCMSC-sEV Isolation and Characterization

For hUCMSC-sEVs isolation, hUCMSCs from the three donors at passage 5 were pooled and cultured until they reached 80% confluency. Once confluency was achieved, the spent culture medium was carefully discarded, and the cells were washed three times with phosphate-buffered saline (PBS) to remove any residual serum or medium components. Subsequently, the cells were incubated in low-glucose Dulbecco’s Modified Eagle Medium (LG-DMEM; Sigma-Aldrich, St. Louis, MO, USA) without serum for 24 h to allow for hUCMSC-sEV production. The conditioned medium was then collected and filtered through a 0.22 µm filter membrane to eliminate cellular debris and other large particles.

#### 2.8.1. Tangential Flow Filtration (TFF)

To further purify hUCMSC-sEVs, while minimizing the risk of contamination with protein aggregates, lipoproteins, or other unwanted impurities, the conditioned medium was centrifuged at 2000× *g* for 15 min followed by TFF. This method used the MINIMATE TFF Capsule fitted with a 100 kDa molecular weight cut-off (MWCO) OMEGA membrane (Pall, New York, NY, USA). Following the completion of the TFF process, the isolated hUCMSC-sEV were carefully resuspended in PBS, aliquoted to avoid repeated freeze–thaw cycles, and stored at −80 °C until further experiments or analyses were performed.

#### 2.8.2. Characterization of hUCMSC-sEVs

The isolation and characterization of hUCMSC-sEVs adhered to the Minimal Information for Studies of Extracellular Vesicles 2023 (MISEV2023) guidelines to ensure reproducibility and reliability. Total protein was quantified using the Pierce™ BCA Protein Assay Kit (Thermo Fisher Scientific™, Boston, MA, USA) according to the manufacturer’s protocol, ensuring standardized measurement procedures. The protein content served as a baseline for the downstream analyses and verification of sEV purity. Western blotting was employed to validate the presence of EV-specific markers, such as CD63 and TSG101, which are conventional markers of EV identity. Simultaneously, the absence of the cellular contaminant GP 96 (an endoplasmic reticulum marker) was evaluated. For intracellular marker analysis, hUCMSC-sEVs were lysed using a radioimmunoprecipitation assay (RIPA) buffer enriched with phenylmethyl sulfonyl fluoride (PMSF) and a protease inhibitor cocktail (Thermo Fisher Scientific) to maintain protein stability during extraction. The extracted proteins were then separated by 10–12% sodium dodecyl sulfate-polyacrylamide gel (SDS-PAGE). After electrophoresis, the proteins were transferred to nitrocellulose membranes for subsequent immunoblotting analysis, enabling the detection of specific intracellular markers. The membranes were incubated with primary antibodies targeting the desired markers, followed by incubation with horseradish peroxidase (HRP)-conjugated secondary antibodies. Signal detection was performed using enhanced chemiluminescence (ECL) substrate, and protein bands were visualized and quantified using a chemiluminescent imaging system. Primary antibodies specific to CD63, TSG101, and GP 96 from Cell Signaling Technology, Danvers, IL, USA, were applied to the membranes, followed by incubation with HRP-conjugated secondary antibodies (Cell Signaling Technology, Danvers, IL, USA). The protein bands were visualized using a chemiluminescent detection system, and images were captured using the Amersham Imager 600 (Cytiva, Marlborough, MA, USA), confirming the identity and purity of the isolated hUCMSC-sEVs.

Nanoparticle Tracking Analysis (NTA) was carried out to determine the size distribution and concentration of the hUCMSC-sEVs. For accurate measurements, the hUCMSC-sEVs samples were diluted 1:1000 in filtered phosphate-buffered saline (PBS) to achieve an optimal particle concentration range (20–200 particles per frame) suitable for the instrument’s sensitivity. Using a Nanosight NS300 instrument (Malvern Panalytical, Misterton, Doncaster, UK) equipped with a 488 nm laser, five 1 min videos were recorded for each sample at 25 °C. The videos were analyzed with the finite track length adjustment (FTLA) algorithm, which tracks the Brownian motion of particles in the suspension, yielding detailed information about the hUCMSC-sEVs size distribution and concentration. In addition, the structural integrity and morphology of the hUCMSC-sEVs were assessed using transmission electron microscopy (TEM). For this, the hUCMSC-sEVs were deposited onto formvar-coated copper grids, ensuring an even distribution, and were negatively stained with 2% phosphotungstic acid (PTA) to enhance the contrast for imaging. The grids were then examined under a LEO Libra-120 TEM.

### 2.9. Primary Human Dermal Fibroblast Isolation and Culture

Primary human dermal fibroblasts (PHDFs) were isolated from human skin samples collected as redundant tissue after surgery from three consenting patients. The skin samples were processed by cutting them into small (1–2 cm) pieces and cleaning them using DPBS. They were digested with 0.6% collagenase Type I (Worthington-Biochemical Corporation) for 4–6 h at 37 °C in a shaker incubator, followed by trypsinization with trypsin-EDTA for 10 min. The cell suspension was centrifuged and resuspended in F12: DMEM (Gibco/BRL) supplemented with 10% fetal bovine serum (FBS) (Gibco). Cells were seeded at 1 × 10^4^ cells/cm^2^ in a 6-well polystyrene culture plate and incubated at 37 °C in 5% CO_2_. The medium was replenished every 2–3 days until the cells achieved 70–80% confluence, at which point differential trypsinization was performed. The fibroblasts were subsequently expanded in a 75 cm^2^ culture flask containing F12: DMEM with 10% FBS. For all experiments, PHDFs between passages 2 to 4 were used. A total of 50,000 cells in 20 µL of culture medium were seeded onto the top of the hydrogels prepared under sterile conditions in a biosafety cabinet.

### 2.10. hUCMSC-sEV Loaded Hydrogel Biocompatibility

To further functionalize the hydrogels, the purified hUCMSC-sEVs were incorporated into the 10% GECL hydrogel. The hUCMSC-sEVs, previously isolated and resuspended in 50 μL of PBS, were added to the 10% GECL solution at various concentrations (125, 100, 75, 50, and 25 μg/mL). The mixture was stirred at 4 °C to ensure a homogeneous distribution of hUCMSC-sEVs throughout the hydrogel matrix.

#### 2.10.1. MTT Assay

This assay was conducted using hydrogel leachates from 10% GECL loaded with hUCMSC-sEVs at concentrations of 125, 100, 75, 50, and 25 µg/mL, according to the ISO 10993-12 protocol [35]. A leachate (2 g/mL) was prepared, and PHDFs were seeded at a density of 5000 cells/cm^2^ in a 48-well plate. After 24 h of incubation at 37 °C in 5% CO_2_, 200 µL of hydrogel leachate at each concentration was added to the respective triplicate wells. The MTT assay (Sigma-Aldrich, St. Louis, MO, USA) was performed to determine cell viability after 48 h. The cells were rinsed with PBS, and 90 µL of pure medium with 10 µL of 5 mg/mL MTT solution was added, yielding a final concentration of 0.5 mg/mL. After 4 h at 37 °C, 87.5 µL of the solution was removed, and 100 µL of DMSO (Sigma-Aldrich, St. Louis, MO, USA) was added to dissolve the formazan. The absorbance was measured using a spectrophotometer (BioTek, Power Wave XS, Highland Park, IL, USA) to calculate cell viability (%) using Formula (5):

Cell Viability (%) = (Ab^t^/Ab^c^) × 100 (5)

where Ab^t^ represents the absorbance of the treatment group, and Ab^c^ represents the control group.

#### 2.10.2. LIVE/DEAD Assay

Cell viability was assessed using the LIVE/DEAD™ Cell (Invitrogen, Waltham, MA, USA). Various concentrations of hUCMSC-sEVs (125, 100, 75, 50, and 25 µg/mL) were incorporated with 10% GECL. PHDFs were seeded onto the hydrogels one day prior and then exposed to calcein-AM and EthD-1 in PBS for 30 min at 37 °C, following the manufacturer’s instructions. Cells were gently washed with PBS and observed under a fluorescence microscope (Nikon A1R-A1, Shinagawa-ku, Tokyo, Japan) at ×100 magnification. Live cells appeared green, while dead cells appeared red.

#### 2.10.3. Cell Attachment Percentage

The cell attachment percentage was evaluated using trypan blue staining. After initial seeding and incubation, unattached cells were collected and visualized using a hemocytometer (Optik Labor, Görlitz, Germany). The cell attachment percentage was calculated using Formula (6):

Cell Attachment (%) = [(Ci − Cd)/Ci] × 100(6)

where Ci Seeded Cell is the initial cell seeding, and Cd is the number of unattached cells in DPBS.

#### 2.10.4. Scratch Wound Assay

The scratch assay was used to assess PHDF migration. To study the wound healing effect of 10% GECL leachate containing sEVs concentrations (75 and 100) µg/mL on PHDFs. The tip of a sterile pipette was used to scratch confluent HDF monolayers in the center of each well. After removing the culture medium, the cells were washed with DPBS (Sigma-Aldrich) and grown in the biomaterial leachate media containing sEVs at concentrations of 0, 75, and 100 µg/mL. The plate was then placed in an incubator, and images were taken every 12 h until the cells migrated to close the scratch, typically within 0 to 72 h. For each of the three biological samples (n = 3), three technical replicates were conducted. A Nikon A1R-A1 live-cell imaging microscope was used to obtain pictures at 1 h intervals to estimate the wound healing closure using Formula (7):

Wound closure (%) = A(t) 100/A (0)(7)

where A (0) is the initial wound area, and A(t) is the wound area at each time point.

The wound closure percentage was calculated using the ImageJ software.

### 2.11. Statistical Analysis

All experiments were conducted with three biological replicates and three technical replicates unless otherwise stated. Results are expressed as mean ± standard deviation (SD). Statistical comparisons were made using one-way or two-way Analysis of Variance (ANOVA) followed by Tukey’s post hoc test for multiple comparisons or an unpaired Student’s *t*-test for comparisons between two groups using GraphPad Prism 9.0 (GraphPad Software, Inc., San Diego, CA, USA). A *p*-value < 0.05 was considered statistically significant.

## 3. Results

### 3.1. Gross Appearance and Polymerization Time

**Figure 2:**
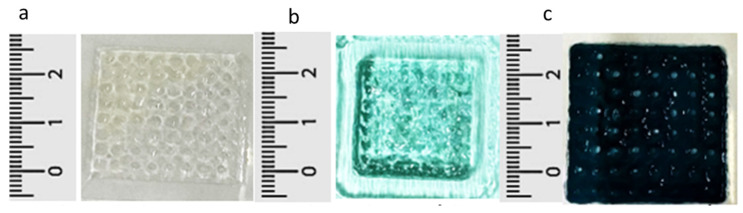
Macroscopic appearance of four-layer 3D-printed hydrogels: top-view images of (**a**) non-crosslinked gelatin hydrogel (GNC), (**b**) crosslinked 8% gelatin hydrogel (8% GECL), and (**c**) crosslinked 10% gelatin hydrogel (10% GECL), illustrating the changes in color and texture following crosslinking with 0.3% genipin (GNP).

GE hydrogels were successfully fabricated, chemically crosslinked using GNPs, and 3D bioprinted via an extrusion-based 3D bioprinter. The GNC hydrogels appeared translucent with a softer texture, which is characteristic of being non-crosslinked. Upon crosslinking, the 8% GECL and 10% GECL exhibited a bluish-green hue and grid-like design with increasing rigidity, confirming the successful formation of the crosslinked GECL hydrogels. These color changes result from the rapid reaction between the GNP and the primary amines in the presence of oxygen, facilitating the crosslinking process, as shown in Figure 2.

**Figure 3:**
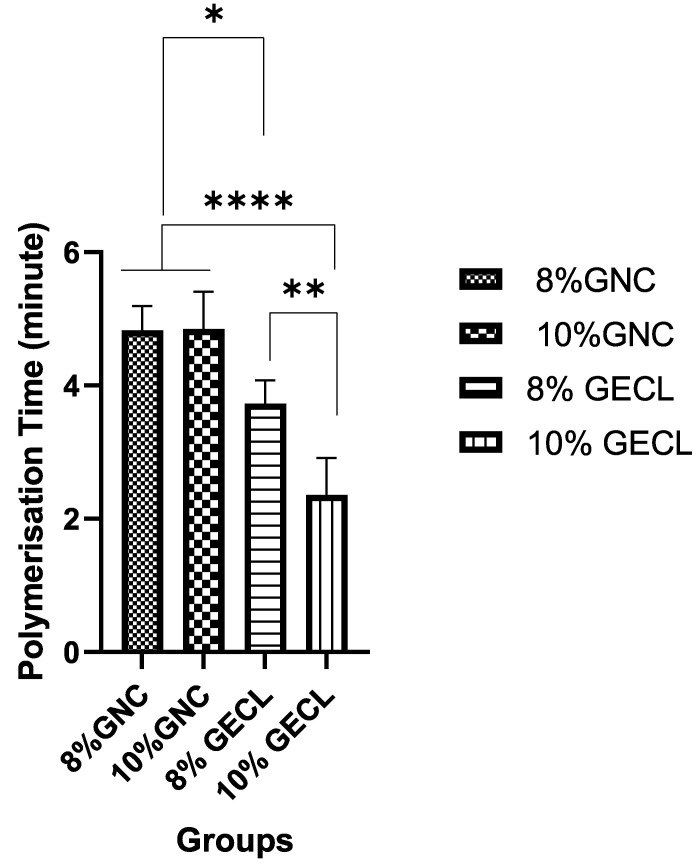
Polymerization time assessment for both non-crosslinked and crosslinked hydrogels. Abbreviations: 8% and 10% gelatin non-crosslinked (8% GNC, 10% GNC) and 8% and 10% gelatin crosslinked (8% GECL, 10% GECL). Data are presented as mean ± standard deviation (n = 5), with statistical significance denoted as * *p* < 0.05, ** *p* < 0.01, and **** *p* < 0.0001.

When assessing the polymerization time, crosslinking significantly reduced the polymerization time compared with the non-crosslinked controls (*p* < 0.0001) (Figure 2) at 22–24 °C. The 8% GNC and 10% GNC polymerization times were 4.83 ± 0.37 and 4.85 ± 0.56 min, respectively. The 8% GECL and 10% GECL polymerization times were 3.73 ± 0.70 and 2.35 ± 0.78 min, respectively, demonstrating that 10% GECL achieved significantly faster polymerization (*p* < 0.01) (Figure 3).

### 3.2. Physical Analysis

**Figure 4:**
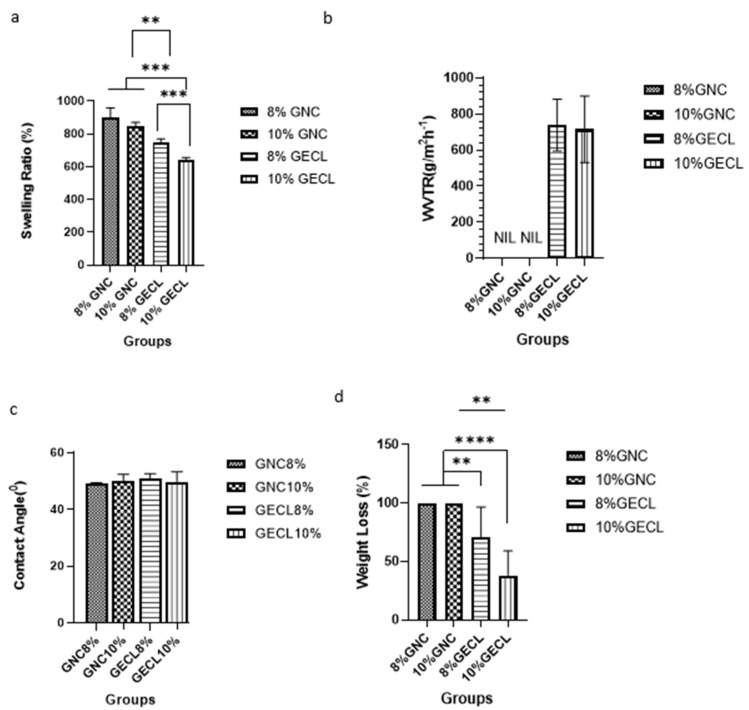
The physical characterization of the gelatin formulations (8% and 10%) was performed in both the crosslinked and non-crosslinked states. The analysis involved the following parameters: (**a**) swelling ratio, (**b**) water vapor transmission rate, (**c**) contact angle, and (**d**) weight loss. Abbreviations used include 8% and 10% gelatin non-crosslinked (8% GNC, 10% GNC) and 8% and 10% gelatin crosslinked (8% GECL, 10% GECL). Data are presented as mean ± standard deviation (n = 3), with statistical significance indicated as ** *p* < 0.01, *** *p* < 0.001, and **** *p* < 0.0001.

The physical properties of the fabricated hydrogels were evaluated for their swelling ratio, porosity, WVTR, contact angle, and biodegradation, with the results presented in Figure 4. Swelling ratio analysis (Figure 4a) showed that non-crosslinked GNC hydrogels had a higher swelling ratio, with 8% GNC and 10% GNC achieving 897.2 ± 61.90% and 843.7 ± 27.37%, respectively. Crosslinked GECL hydrogels exhibited optimal swelling (500–800%), with 8% GECL at 784.5 ± 22.13% and 10% GECL at 638.2 ± 18.36%, and the difference between the two groups was significant (*p* < 0.001).

The moisture retention capacity of the different groups was assessed by measuring the water vapor transmission rate (WVTR), as illustrated in Figure 4b.

The 8% and 10% GECL formulations scored WVTR of 738.2 ± 143.6 g/m^2^/h and 715.9 ± 184.7 g/m^2^/h, respectively, demonstrating no significant difference between the two groups. The wettability of the hydrogels was measured via a contact angle test. The angle values of the groups are shown in Figure 4c, which revealed that all hydrogels exhibited angles below 90°, indicative of hydrophilic surfaces, which are critical for cell attachment. The 8% GNC and 10% GNC had the lowest contact angles (49.08 ± 0.36° and 49.49 ± 2.43°, respectively), lower than the crosslinked GECL hydrogels, which displayed slightly higher angles (8% GECL: 50.65 ± 1.90°; 10% GECL: 49.74 ± 3.56°). Enzymatic biodegradation was assessed by quantifying the percentage of weight loss after 24 h of incubation, providing insights into the hydrogel’s structural stability and degradation profile under physiological conditions. The analysis revealed that there is a significant difference between the crosslinked and non-crosslinked groups, particularly between 8% GNC vs. 8% GECL (*p* < 0.01) and between 10% GNC vs. 10% GECL (*p* < 0.0001). The 8% and 10% GNC completely degraded, scoring 100% weight loss. The mean weight loss for 8% GECL is 70.83 ± 25.73%, showing moderate degradation, while 10% GECL had the lowest degradation (38.12 ± 20.82%).

**Figure 5:**
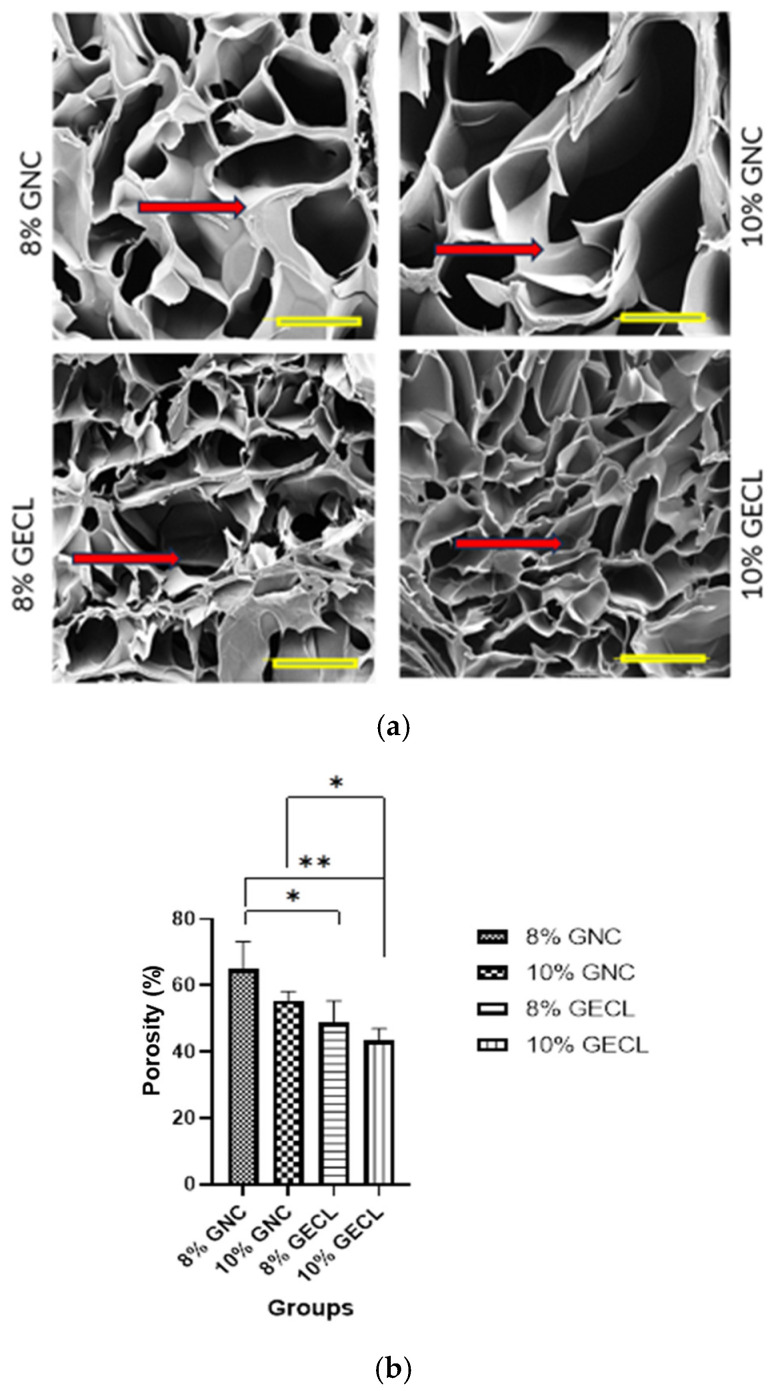
(**a**) Microporous structure visualization by scanning electron microscopy of the gelatin hydrogel (cross-sectional view) at 200× magnification. The red arrow indicates the interconnected pores. Each yellow scale bar represents 20 μm. (**b**) The porosity percentage of the groups using the liquid displacement method. Abbreviations: 8% and 10% gelation non-crosslinked (8% and 10% GNC), 8% and 10% gelatin crosslinked (8% GECL, 10% GECL), N = 4, n = 5, * *p* < 0.05, ** *p* < 0.01.

The SEM micrographs of the 8% GNC, 10% GNC, 8% GECL, and 10% GECL groups revealed a heterogeneous porous structure in all groups, as shown in the cross-sectional images in Figure 5a. The porosity is demonstrated in Figure 5b, and the results for each group, presented as mean ± SD, are as follows: 8% GNC (64.95 ± 8.255%), 10% GNC (55.46 ± 2.707%), 8% GECL (48.68 ± 6.682%), and 10% GECL (43.45 ± 3.544%). These results indicate that the GNC formulations exhibit higher porosity compared to the GECL formulations, with the porosity decreasing as the concentration increases within both groups.

### 3.3. Chemical Characterization

**Figure 6:**
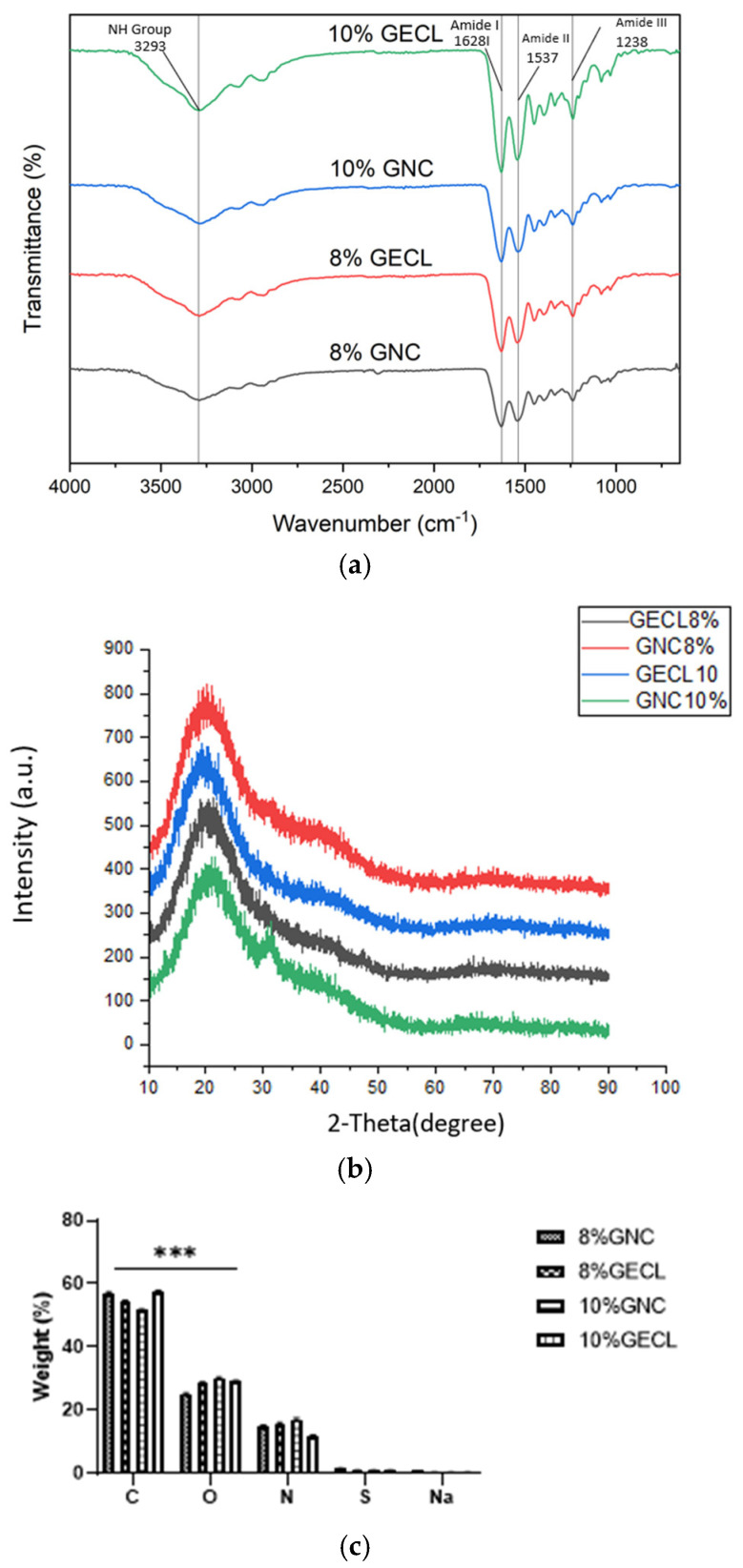
Chemical characterization of the hydrogel. (**a**) FTIR spectra of 8% and 10% GECL; 8% and 10% GNC and fabricated hydrogels. (**b**) X-ray diffraction analysis (XRD) of hydrogel. (**c**) Element distribution of carbon, oxygen, and nitrogen in the genipin crosslinked and non-crosslinked gelatin hydrogels. Abbreviations: 8% and 10% gelation non-crosslinked (8% and 10% GNC), 8% and 10% gelatin crosslinked (8% and 10% GECL), two-way ANOVA, *** *p* < 0.001.

The FTIR spectra confirmed the presence of key functional groups in the hydrogel formulations, with a broad peak at 3293 cm⁻^1^ indicating NH stretching vibrations. The characteristic amide peaks were at 1628 cm⁻^1^ (amide I, C=O stretching), 1537 cm⁻^1^ (amide II, NH bending and CN stretching), and 1238 cm⁻^1^ (amide III, CN stretching and NH bending). The FTIR spectra also showed slight differences between GNC and GECL in terms of peak intensity and molecular interactions. GECL samples (both 10% and 8%) exhibited higher peak intensities at the amide I (1628 cm⁻^1^) and amide II (1537 cm⁻^1^) regions compared to GNC (Figure 6a).

The XRD analysis revealed the degree of crystallinity and amorphous characteristics of the GECL and GNC hydrogels (Figure 6b). The observed crystallinity percentages of 8% GECL (16.99%) and 10% GECL (15.4%) indicate slightly higher crystalline regions compared to 8% GNC (13.6%) and 10% GNC (14.8%). The amorphous nature of these hydrogels dominates, with values exceeding 80%. The intensity plot further supports these findings, showing broad peaks centered around 2θ ≈ 28°, characteristic of the amorphous structure. A minor sharp peak is visible between 30° and 40°, which correlates with the crystalline contribution from the GNPs within the hybrid hydrogels.

The elemental composition of the hydrogels was analyzed using EDX spectroscopy, as detailed in Table 1. Carbon (C) was primarily associated with the gelatin polymers, while oxygen (O), nitrogen (N), sulfur (S), and sodium (Na) were also detected. Nitrogen levels remained consistent across all samples, while sulfur and sodium were present in trace amounts. The analysis showed that carbon was the predominant element, with the following percentages: 57.8 ± 0.4% for 8% GNC, 54.5 ± 0.3% for 8% GECL, 57.5 ± 0.3% for 10% GNC, and 51.6 ± 0.3% for 10% GECL. Oxygen was the second most abundant element, with values of 25.2 ± 0.3% for 8% GNC, 28.6 ± 0.3% for 8% GECL, 29.2 ± 0.2% for 10% GNC, and 30.2 ± 0.3% for 10% GECL, as shown in Figure 6c.

### 3.4. Mechanical Testing Analysis

**Figure 7:**
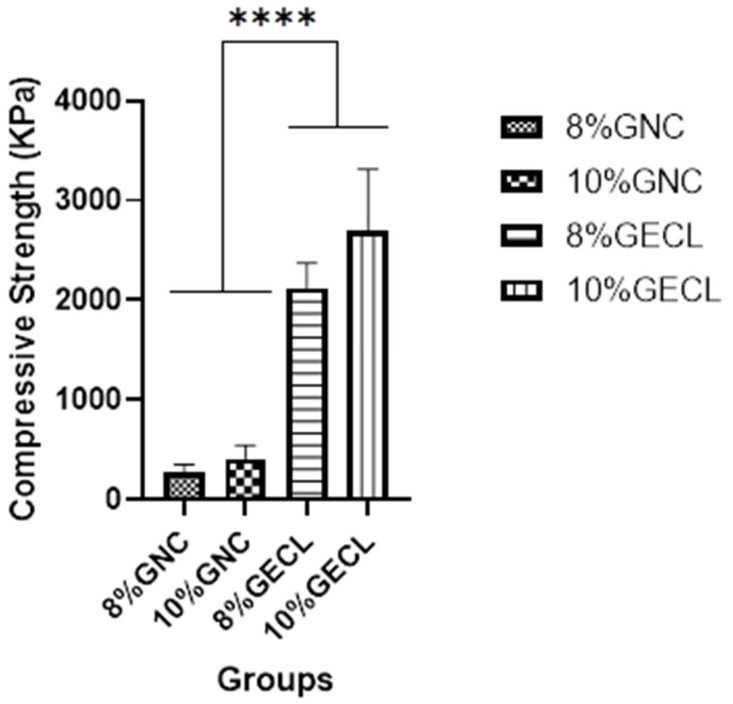
Mechanical property analysis of different hydrogels. Abbreviations: 8% and 10% gelation non-crosslinked (8% and 10% GNC), 8% and 10% gelatin crosslinked (8% and 10% GECL), two-way ANOVA, **** *p* < 0.0001.

Compression testing, shown in Figure 7, revealed that the crosslinked hydrogels (8% and 10% GECL) exhibited significantly higher compressive strength compared to the non-crosslinked (GNC) hydrogels. The 10% GECL hydrogel showed the highest compressive strength at 2695 ± 621.2 kPa, followed by the 8% GECL at 2125 ± 246.9 kPa. In contrast, the non-crosslinked hydrogels (10% GNC: 410.8 ± 133.7 kPa and 8% GNC: 274.5 ± 75.9 kPa) demonstrated much lower strength. These results highlight the significant role of genipin crosslinking in enhancing the mechanical properties of the hydrogels, making them suitable for use in dynamic biological applications.

### 3.5. hUCMSC Isolation and Characterization

**Figure 8:**
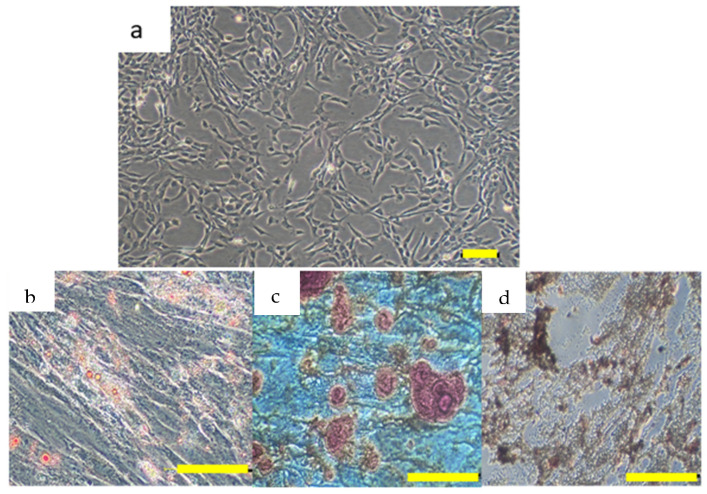
(**a**) P5 hUCMSCs exhibit a fibroblastic spindle-shaped morphology and adhere to the plastic surface. Magnification: 40×; scale bar: 100 μm. Trilineage differentiation potential of P5 hUCMSCs demonstrated through osteogenic, chondrogenic, and adipogenic differentiation: (**b**) Adipogenic differentiation confirmed by lipid droplet formation (Oil Red O staining); magnification: 200×. (**c**) Osteogenic differentiation confirmed by calcium deposition (Alizarin Red staining); magnification: 40×. (**d**) Chondrogenic differentiation confirmed by proteoglycan accumulation (Safranin O staining); magnification: 100×. Scale bar: 100 μm. Abbreviations: passage 5 (P5); human umbilical cord mesenchymal stromal cells (hUCMSCs).

hUCMSCs were successfully isolated, expanded, and characterized at P5 in accordance with the criteria established by ISCT [34]. The cells adhered to the plastic surfaces and exhibited a spindle-shaped morphology, which is typical of hUCMSCs (Figure 8a). Flow cytometry analysis of hUCMSCs revealed that the cells tested positive for MSC markers, including CD73, CD90, and CD105 (96.76%, 99.98%, and 99.85%, respectively), and the absence of hematopoietic markers such as CD45, CD34, CD11B, CD19, and HLA (<2%). Trilineage differentiation assessment demonstrated the differentiation potential into adipogenic, osteogenic, and chondrogenic lineages (Figure 8b–d). For adipogenic differentiation, Oil Red O staining revealed lipid droplet accumulation, confirming their capacity to develop into adipocytes. Osteogenic differentiation was verified through Alizarin Red staining, which highlighted calcium deposits indicative of bone-like structure formation. Similarly, chondrogenic differentiation, assessed by Safranin O staining, showed the formation of a cartilage matrix, demonstrating the cells’ ability to differentiate into chondrocytes.

### 3.6. hUCMSC-sEV Isolation and Characterization

**Figure 9:**
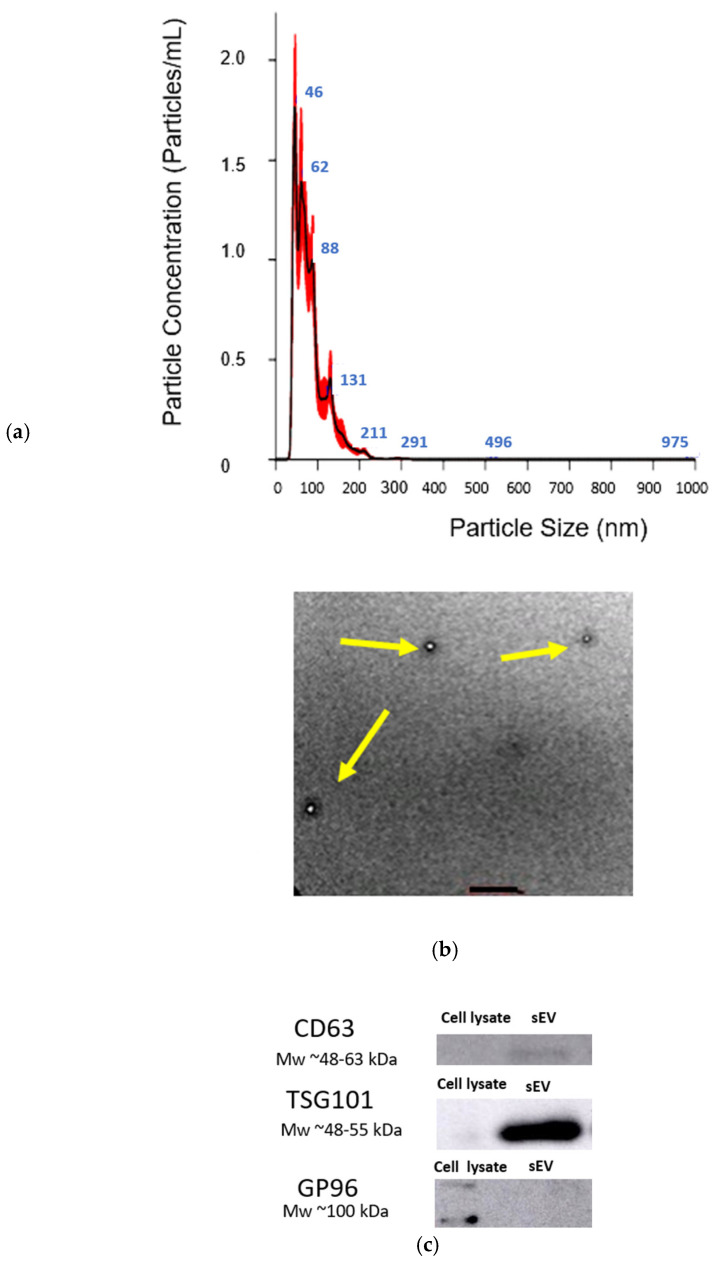
Characterization of hUCMSC-sEVs: (**a**) NTA measurement confirms the presence of hUCMSC-sEVs with the size range of 50–200 nm. (**b**) TEM image of hUCMSC-sEVs, confirms the presence of vesicles with diameters less than 200 nm. The arrow highlights a representative hUCMSC-sEVs exhibiting the characteristic spherical morphology and size consistent with sEVs. Magnification = ×40 K. Scale bar (black): 500 nm. (**c**) hUCMSC-sEVs express CD63 and TSG101 (positive markers), whereas they lack GP96 expression (negative markers). Abbreviations: human umbilical cord mesenchymal stromal cell-derived small extracellular vesicles (hUCMSC-sEVs); Nanoparticle Tracking Analysis (NTA); transmission electron microscopy (TEM). (TEM) image of hUCMSC-sEVs. The arrow indicates an exosome with spherical morphology and a diameter of less than 200 nm.

hUCMSC-sEVs were isolated via centrifugation and TFF. Figure 9a presents the NTA results, revealing an average particle size of 40.7 ± 2.2 nm and a concentration of 8.91 × 10^8^ particles/mL. The size distribution of the hUCMSC-sEVs ranged between 50 nm and 200 nm, consistent with the expected range for sEVs. hUCMSC-sEV protein quantification using the BCA assay revealed a protein concentration of 2047.088 μg/mL. TEM analysis further demonstrated that the hUCMSC-sEVs exhibited a spherical morphology with a size of less than 200 nm (Figure 9b). Meanwhile, Western blot analysis confirmed the expression of key sEV markers CD63 (48–63 kDa) and TSG101 (48–55 kDa), while the negative marker GP 96 (100 kDa), indicative of cellular contamination, was absent in the hUCMSC-sEV samples but present in the cell lysate (Figure 9c). These findings validated the successful isolation of hUCMSC-sEV using the TFF system.

### 3.7. Biocompatibility of the Hydrogels

Based on the previous results, the hydrogel 10% GECL was selected among all formulations to proceed with cell biocompatibility testing as it achieved an optimal polymerization time and biodegradation, besides achieving a suitable swelling ratio, WVTR, porosity, and wettability. To further investigate its biocompatibility, hUCMSC-sEVs suspended in PBS were incorporated into the 10% GECL solution at various concentrations (125, 100, 75, 50, and 25 μg/mL).

**Figure 10:**
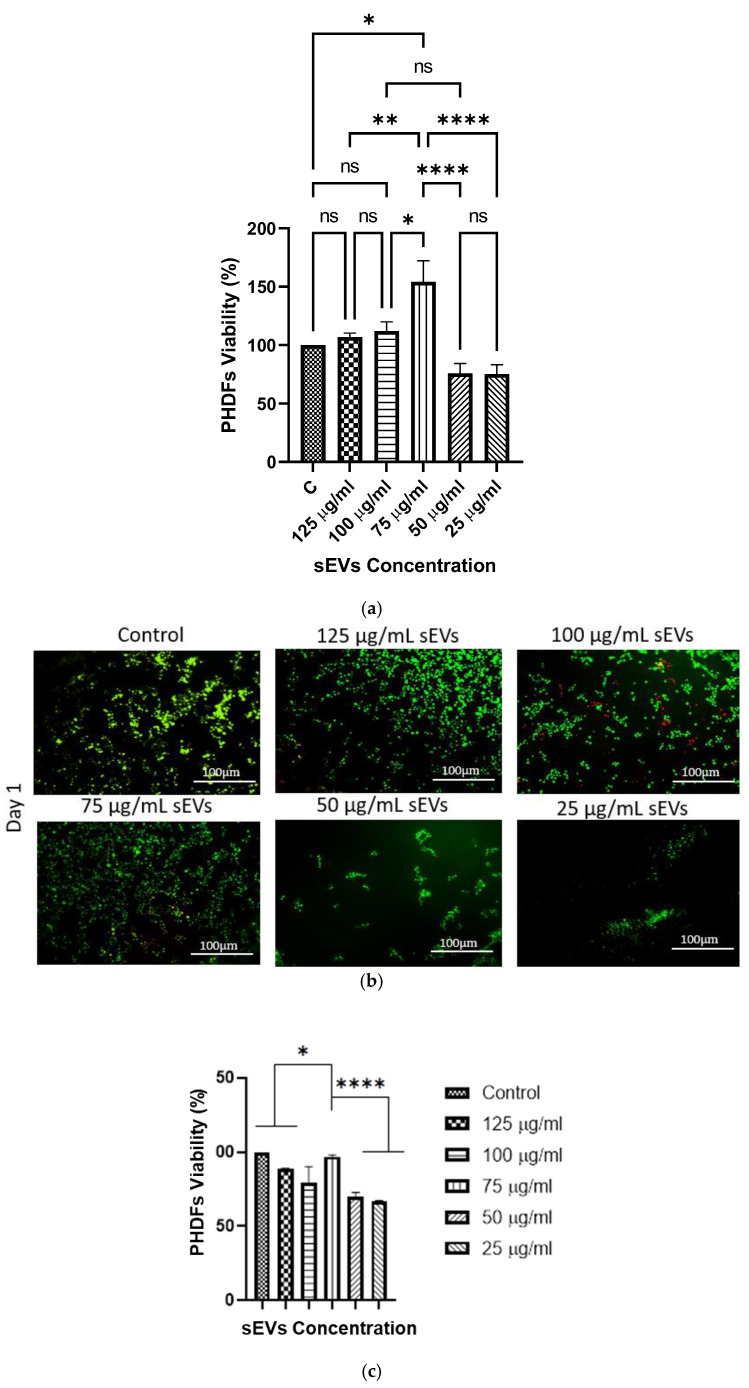

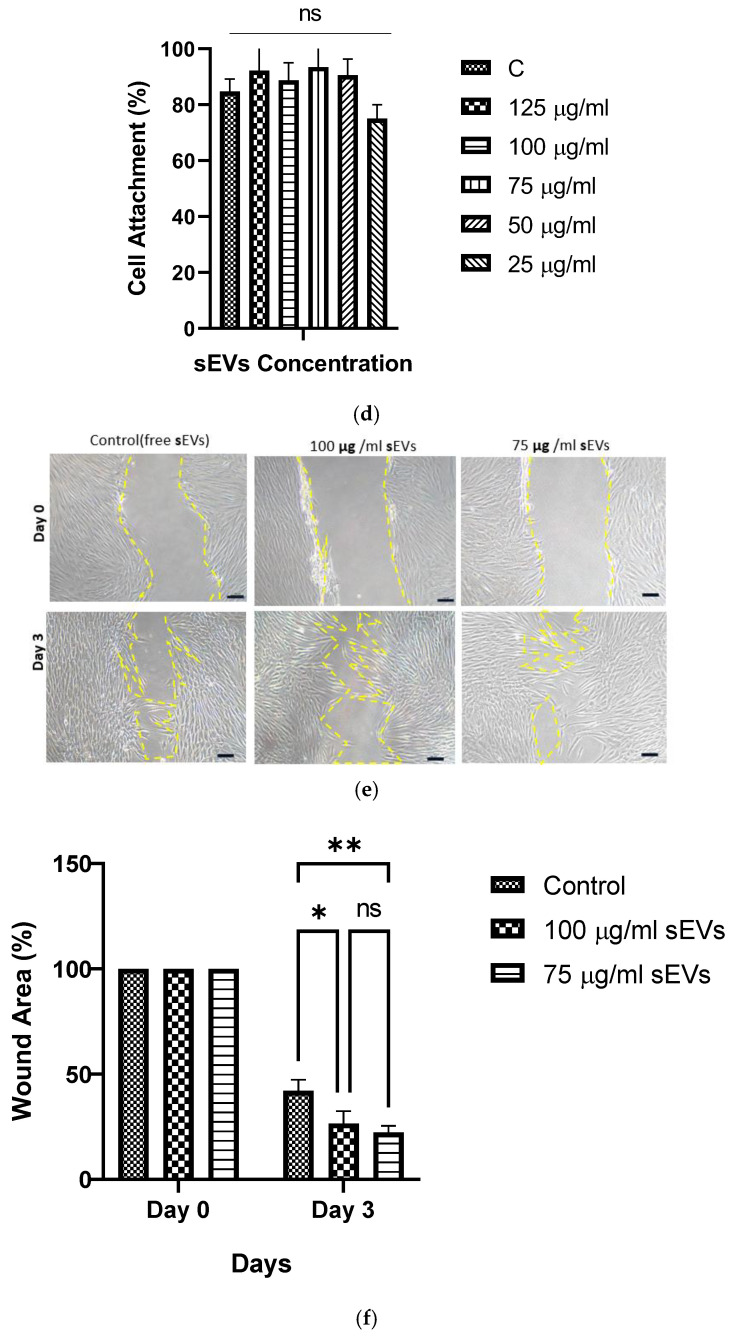
The biocompatibility evaluation of various concentrations of human umbilical cord mesenchymal stromal cell-derived small extracellular vesicles (hUCMSC-sEVs) (125, 100, 75, 50, and 25 μg/mL) was conducted using primary human dermal fibroblasts (PHDFs) incorporated within a 10% gelatin-crosslinked hydrogel (10% GECL). (**a**) The assessment included the following: (**a**) cell viability (%) analysis using the MTT assay at 48 h; (**b**) LIVE/DEAD assay at 24 h, with representative fluorescent images showing live (green) and dead (red) cells seeded on hydrogels, indicating cell viability and cytocompatibility; (**c**) the cell viability quantification of human dermal fibroblasts using LIVE/DEAD assay; (**d**) the quantification of PHDF attachment (%) at 24 h; imaging was performed at 100× magnification, with a scale bar of 100 μm; (**e**) wound scratch assay of HDFs using leachate media containing human umbilical cord mesenchymal stromal cell-derived small extracellular vesicles (hUCMSC-sEVs) (100 and 75 μg/mL) for 72 h (scale bar, 100 μm); (**f**) healing progression for wound scratch assay. Imaging was performed at 100× magnification, with a scale bar of 100 μm. Data are presented as mean ± standard deviation (N = 3, n = 3), “ns” denotes “no significant difference” between groups.The yellow dashed line highlights the area reduction for comparison across different conditions.Thank you for your valuable feedback. with statistical significance denoted as * *p* < 0.05, ** *p* < 0.01, and **** *p* < 0.0001.

The biocompatibility of hUCMSC-sEVs loaded into 10% GECL with cultured PHDFs was significantly influenced by the concentration of hUCMSC-sEVs loaded into 10% GECL when tested using an MTT assay (Figure 10a). Statistical analysis confirmed that 10% GECL incorporated with hUCMSC-sEVs at a concentration of 75 μg/mL resulted in the most significant increase in cell viability (154.3 ± 40.1%), outperforming the control group (C = 10% GECL without hUCMSC-sEVs) that was set at 100%. The viability of PHDFs at the other hUCMSC-sEVs concentrations were as follows: 125 µg/mL (106.7 ± 9.4%), 100 µg/ML (112.0 ± 17.7%), 50 µg/mL (75.7 ± 22.7%), and 25 µg/mL (75.3 ± 18.0%).

In addition, cell viability was evaluated using a live/dead assay after 24 h of incubation, as illustrated in Figure 10b. Fluorescent imaging of live (green) and dead (red) PHDFs revealed that cells were uniformly distributed over the 3D hydrogel structure without obvious morphological abnormalities, as cells appeared round, with all concentrations. The results demonstrated differences in cell distribution and viability across various hUCMSC-sEV concentrations. Quantitatively, the percentage of live cells was the highest among the hydrogel sEV treatment groups; 75 µg/mL exhibited the highest percentage of cell viability (96.98 ± 56.86%), followed by 125 µg/mL (88.54 ± 0.495%), 100 µg/mL (79.72 ± 10.61%), 50 µg/mL (69.63 ± 3.359%), and 25 µg/mL (67.06 ± 0.275%), as illustrated in Figure 10c. Corresponding to the MTT results, the 75 µg/mL hUCMSC-sEVs loaded into 10% GECL demonstrated the best viability. In contrast, 50 and 25 µg/mL hUCMSC-sEVs resulted in lower viability, with sparse green fluorescence and poor cell attachment; 125 µg/mL and 100 µg/mL demonstrated enhanced viability compared to the control, 50 and 25 µg/mL, but they were lower than 75 µg/mL.

Assessing PHDF attachment, excellent adherence was observed in all 10% GECL hydrogels on day 1, with the attachment percentages ranging from 80% to 90%, regardless of the hUCMSC-sEV concentration (C, 125, 100, 75, 50, and 25 μg/mL). Quantitative analysis of cell attachment revealed the following percentages: 84.67 ± 4.51%, 92.17 ± 10.54%, 88.67 ± 6.35%, 93.43 ± 6.65%, 90.50 ± 5.77%, and 75.00 ± 5.00% for C, 125, 100, 75, 50, and 25 μg/mL, respectively, with no statistically significant differences. An in vitro scratch wound healing assay was performed to evaluate the migration activity of the HDFs for future wound healing. As shown in Figure 10d, treatment with UC-MSC-derived small extracellular vesicles (sEVs) at both 100 μg/mL and 75 μg/mL significantly promoted cell migration after 72 h of exposure. Notably, the fibroblast monolayer treated with sEVs demonstrated complete wound closure by 72 h, in stark contrast to the untreated control.

Quantitative analysis of the wound area confirmed this observation: cells treated with 100 μg/mL sEVs exhibited a reduced wound area of 26.62% ± 5.30, while the 75 μg/mL sEV group showed an even lower wound area of 22.28% ± 2.92. In comparison, the control group maintained a significantly larger wound area of 42.01% ± 5.26.

## 4. Discussion

The hUCMSC-EVs, known for their regenerative potential, deliver key bioactive signals that support tissue repair, reduce inflammation, stimulate angiogenesis, and enhance cell regeneration [10]. However, for the complete regeneration of large wounds, a 3D scaffold is essential to support cell attachment, migration, and proper tissue formation. Hydrogels provide this structural support while maintaining hydration and allowing the controlled release of hUCMSC-EVs. This slows their degradation and clearance, extending their therapeutic effects [12]. Additionally, advances in 3D printing technology allow for the design of personalized hydrogel scaffolds tailored to the unique shape and size of individual wounds, improving treatment precision and efficacy [29]. The combination of hUCMSC-sEVs with bioengineered hydrogels offers a promising personalized approach for treating chronic and complex wounds.

Hence, this study aimed to develop and characterize 3D bioprinted gelatin–genipin (GNP)-crosslinked hydrogels loaded with hUCMSC-EVs for future skin wound healing applications. Initially, we formulated and optimized GNP-crosslinked gelatin hydrogels using four different combinations of gelatin concentrations, with and without GNP. The formulations included non-crosslinked gelatin hydrogels (GNC and 10% GNC, where gelatin concentration is expressed in *w*/*v*) and GNP-crosslinked hydrogels (8% GECL and 10% GECL, containing 0.3% GNP). Each hydrogel type was evaluated for its physicochemical properties to determine its potential for wound healing applications.

A key advantage of this hydrogel system is its sustainability and biocompatibility, as both gelatin and GNP are naturally derived eco-friendly biomaterials with well-documented therapeutic potential. Gelatin, sourced from various waste materials, not only provides a cost-effective and biodegradable alternative but also mimics the ECM, facilitating cell adhesion and tissue regeneration [36,37]. Likewise, GNP, a natural crosslinker, offers a green alternative to synthetic chemical crosslinker, enhancing the hydrogel’s mechanical properties while preserving high biocompatibility [26]. These attributes make gelatin–GNP hydrogels a promising platform for wound healing applications, combining structural support with bioactive properties in a commercially viable and environmentally responsible manner.

The physicochemical properties of the hydrogels were evaluated to determine their suitability for both 3D bioprinting and potential wound healing applications. Among these, the polymerization time is a critical parameter influencing the practicality of the 3D printing process [38]. In this study, polymerization occurred within two to five minutes at room temperature (22–24 °C), with the rate significantly improving as gelatin concentration increased. Notably, the hydrogel with 10% gelatin content (10% GECL) demonstrated the fastest polymerization, solidifying in approximately two minutes. This timeframe is considered optimal for 3D bioprinting, offering sufficient working time for precise deposition and structural fidelity, while ensuring rapid solidification post-printing. The fast gelation not only facilitates handling and positioning during clinical application but also supports the maintenance of construct integrity throughout the process.

Previous studies have identified the optimal gelation times for extrusion-based 3D bioprinting. For instance, a study using GNP hybrid gelatin–PVA hydrogels found three minutes to be ideal at 23 °C [39]. In contrast, a similar study found that longer polymerization times of 4–5 min were less favorable, as they delayed solidification, compromising the efficiency of the process.

The bluish-green color change observed during the polymerization process in the GNP hydrogels indicated successful crosslinking. The hydrogels’ appearance revealed clear, translucent, non-crosslinked (GNC) gelatin hydrogels, whereas the crosslinked (GECL) hydrogels displayed a bluish-green hue. This color change is attributed to the reaction of GNPs with primary amines in the presence of oxygen, resulting in the formation of water-soluble blue pigments, as previously reported by Zawani et al. 2022 [40]. Keeping the wound area hydrated is essential for optimal healing, as it prevents eschar formation and supports cell communication through signaling molecules. A moist environment enhances epithelial cell migration and collagen synthesis by fibroblasts and promotes the autolytic debridement of necrotic tissue [41]. To assess the hydration ability of the hydrogel in maintaining a moist wound environment and exudate clearance, several key properties were tested, such as the swelling ratio, porosity, WVTR, and hydrophilicity.

The swelling ratio is a paramount parameter that quantifies a hydrogel’s ability to absorb and retain water, directly reflecting its capacity to maintain a hydrated environment—an essential factor in promoting tissue regeneration and wound healing. A high swelling ratio not only indicates superior absorption of wound exudates but also correlates with improved hydrogel performance as a moisture-retentive dressing. Furthermore, swelling behavior provides valuable insights into the structural integrity and crosslinking efficiency of the hydrogel as well as its potential for sustained drug delivery—both of which are pivotal to its therapeutic efficacy [42]. In this study, all hydrogel formulations exhibited a swelling ratio exceeding 500%, affirming their suitability for wound healing. Notably, non-crosslinked hydrogels showed a higher swelling ratio, likely due to the absence of additional bonding restricting water uptake. Consistent with previous findings, the degree of crosslinking plays a crucial role in modulating swelling behavior, where a denser network may reduce water absorption while still maintaining adequate hydration to support cellular activities essential for tissue repair [43]. However, the tested crosslinked hydrogels still maintained an optimal swelling ratio, striking a balance between excessive swelling, which could lead to hydrogel deformation, and controlled swelling, which ensures moisture retention and blood and body fluid absorption [42].

The variation in the swelling ratio was reflected in the porosity differences between the crosslinked and non-crosslinked hydrogels. As expected, crosslinking reduced the overall porosity [40,44]. The crosslinked hydrogels exhibited an interconnected pore network, which is vital for allowing efficient water absorption and retention within the hydrogel matrix. Beyond its influence on swelling behavior and water infiltration, this porous structure is also essential to support cell infiltration, facilitate the exchange of oxygen and nutrients, and ultimately, accelerate the healing process [45]. This has been reported by another study that also showed reduced porosity due to GNP crosslinking; however, this reduction did not affect the viability of the cells cultured within the hydrogels [46].

Given that porosity directly influences water permeability, it was essential to evaluate the water vapor transmission rate (WVTR), which represents the rate at which water vapor diffuses through the hydrogel matrix [40]. This parameter is critical in assessing the hydrogel’s ability to maintain a moist wound environment—an important factor for effective wound healing. An optimal WVTR helps sustain the ideal moisture balance at the wound site, which is known to facilitate cell migration, enhance collagen synthesis, and minimize the risk of infection. Previous studies have reported that the ideal WVTR for skin biomimetic scaffolds ranges from 700 to 1200 g/m^2^/h, providing a balance between moisture retention and the prevention of wound bed dehydration.

Surface wettability is another measure of hydrogel hydrophilicity, which is important to assess a hydrogel’s suitability for wound healing. A lower contact angle reflects higher hydrophilicity, indicating better cell attachment, moisture retention, and lesion exudate absorption, thus supporting effective healing [47,48]. Additionally, wettability influences the hydrogel’s adhesion capability, as higher wettability enhances adherence to wet biological tissues by reducing the resistance from physiological fluids [48,49]. All the hydrogels in this study demonstrated high hydrophilicity. While crosslinking modifications enhance wettability [50], our findings revealed that GNP only trivially modified the inherently high wettability of the gelatin-based hydrogels. Overall, both the crosslinked and non-crosslinked hydrogels exhibited suitable hydrophilicity for a conducive environment for tissue regeneration.

In vitro biodegradation was evaluated to address the limitation of rapid gelatin degradation post-implantation. Crosslinked hydrogels (GECL) exhibited greater durability than gelatin-only hydrogels, meeting the requirement of at least 14 days for wound healing applications [51]. The 10% GECL showed better stability compared with 8% GECL, apparently due to increased gelatin concentration. GNP crosslinking has been shown to enhance the stability of hydrogels by preventing enzymatic degradation [52]. Rapid degradation could lead to premature scaffold loss before new skin tissue formation occurs [52]. Furthermore, the crosslinker GNP has been reported to improve scaffold stability while providing antioxidant properties to support wound healing and sustain cell migration and differentiation from surrounding tissues [26]. Given their enhanced durability and bioactive properties, crosslinked hydrogels (GECL) present a promising platform for hUCMSC-sEV incorporation and future wound healing applications.

The chemical characterization of GECL and GNC via EDX revealed only minimal elemental changes following the addition of GNP. Gelatin, rich in amino acids and composed of carbon, oxygen, nitrogen, sulfur, and sodium, maintains key functional groups such as carboxylic, amide, ester, aromatic, and alkane [53]. FTIR analysis showed no significant peak shifts between GECL and GNC; however, subtle differences in the intensity and shape of the amide I and II bands indicate that GNP crosslinking induces minor structural modifications in the gelatin matrix. This observation is consistent with earlier findings [51] and another previous study by our research team reporting that genipin and PVA incorporation do not significantly alter the native amorphous structure of gelatin hydrogels [54]. While FTIR provides important insight into functional group interactions, we acknowledge its limitations in resolving fine chemical changes. In future work, we aim to complement these results with more sensitive techniques.

Moreover, XRD results demonstrated that, while GNP incorporation marginally increased the degree of crystallinity, the hydrogels remained predominantly amorphous—an advantageous feature for biomedical applications [55]. Excessive crystallinity in biomaterials has been associated with increased stiffness, which can hinder cellular adhesion and proliferation [56]. The mechanical strength of biomaterials is critical for their ability to withstand pressure during implantation at wound sites [57].

The mechanical properties of the hydrogels, particularly compressive strength, underscore their potential for biological applications. The GNP-crosslinked gelatin hydrogel exhibited enhanced mechanical stability, closely resembling the biomechanical characteristics of native skin, thereby making it well suited for skin-related applications. The incorporation of GECL contributed to a notable increase in compressive strength, reaching approximately 2 MPa. This enhancement is attributed to the stabilizing interactions between GECL and gelatin molecules, which reinforce the hydrogel matrix and improve its structural integrity. Comparable improvements have been observed in other crosslinked systems, such as chitosan composite hydrogels (CS-CGO), where compressive stress increased from 1.9 MPa to 4.2 MPa, further supporting the role of crosslinking in optimizing hydrogel performance [58]. Overall, the improved mechanical properties position these hydrogels as promising candidates for dynamic and mechanically demanding applications, including wound healing and skin regeneration.

In summary, the incorporation of GNPs into the gelatin matrix induced only subtle structural modifications without significantly altering its overall chemical composition or amorphous nature. This balance is critical for wound healing applications, as the maintained amorphous structure facilitates optimal cell infiltration and nutrient diffusion, while the minor enhancements in crystallinity and crosslinking contribute to improved mechanical stability and controlled degradation [58]. Collectively, these properties support a conducive environment for tissue regeneration and suggest that GECL hydrogels are well suited for use in wound healing therapies.

Functionalizing hydrogels with bioactive compounds has been explored to enhance their regenerative properties, such as angiogenesis promotion, immunomodulation, and cell proliferation stimulation [59]. In this context, hUCMSC-sEVs have emerged as a promising tool for hydrogel functionalization because they inherently exhibit these therapeutic effects [60]. The selection of hUCMSCs as an EV source offers several advantages. Their high proliferation capacity, immunomodulatory effects, and robust paracrine signaling contribute significantly to the therapeutic potential of the EVs they release. As key mediators that propagate the beneficial properties of their parent cells [6], hUCMSC-sEVs are expected to deliver these effects to target tissues [61]. Additionally, the non-invasive and ethically favorable process of obtaining hUCMSCs further supports their utility in both clinical and research applications [62].

The characterization of the isolated hUCMSCs was performed according to the ISCT criteria, which are critical for establishing the MSC identity [34]. The hUCMSCs exhibited the characteristic properties of MSCs, such as plastic adherence, spindle-shaped morphology, and the expression of the surface markers CD73, CD90, CD105, and CD44, while being negative for the hematopoietic markers CD34 and CD45 [63]. These findings agree with previous studies that reported similar profiles for MSCs derived from various tissues, including bone marrow and adipose tissue, supporting the claim of the successful isolation of hUCMSCs [64]. The multilineage differentiation potential of adipogenic, osteogenic, and chondrogenic lineages further corroborates the stemness of the isolated cells [65]. Similarly, this comprehensive characterization aligns with our previous findings, confirming the identity of the cells [66].

hUCMSC-sEVs were successfully isolated and characterized using TFF, a highly efficient and scalable technique for EV isolation, particularly suitable for processing large volumes of conditioned medium. Unlike conventional dead-end filtration, TFF directs the fluid tangentially across the membrane surface rather than perpendicularly, reducing the accumulation of retained material and minimizing membrane clogging. This tangential flow mechanism enhances the separation efficiency and ensures consistent performance.

The characterization of hUCMSC-sEVs confirmed their compliance with the criteria outlined in MISEV 2023 [67]. The isolated hUCMSC-sEVs exhibited a round morphology and a size range of 50–200 nm and expressed the canonical EV markers CD63 and TSG101, validating their identity as sEVs [68,69]. The absence of the GP96 marker further indicated the purity of the preparation, confirming minimal cellular contamination [70]. These findings establish the successful isolation and enrichment of hUCMSC-sEVs.

Based on the prior findings, the hydrogel formulation of 10% GECL was chosen for further cell biocompatibility testing. This selection was made due to its optimal polymerization time and slower biodegradation, along with its ideal swelling ratio, WVTR, porosity, and wettability. These characteristics collectively indicate its suitability for biomedical applications.

The biocompatibility of the 3D bioprinted hUCMSC-sEV-incorporated gelatin hydrogel 10% GECL was evaluated by assessing both PHDF viability and attachment. Compared to the hydrogels without hUCMSC-sEV, those incorporating hUCMSC-sEVs exhibited an additive effect on PHDFs viability. This finding indicates that the presence of hUCMSC-sEVs promotes fibroblast survival and proliferation without inducing cytotoxic effects, consistent with previously reported studies highlighting the pro-regenerative and cytocompatibility nature of sEV-based therapies [10,18]. Notably, the concentration of 75 μg/mL of hUCMSC-sEV resulted in significantly higher PHDF viability. Increasing the dose beyond 75 μg/mL led to a decline in PHDFs viability, highlighting the importance of optimizing hUCMSC-sEV concentration for optimal outcomes [71]. Additionally, high PHDF attachment (80–90%) across all hUCMSC-sEVs concentrations further confirmed the excellent biocompatibility of the hydrogel platform. The hUCMSC-sEV-incorporated 10% GECL hydrogels exhibited high cell attachment, likely due to the combined influence of the hydrogel’s improved structure with crosslinking, which provides robust mechanical support for cell adhesion, and the bioactive properties of hUCMSC-sEV. These results underline the potential of 10% GECL hydrogels, particularly those incorporating 75 µg/mL hUCMSC-sEV, as biocompatible scaffolds for tissue engineering and wound healing. Meanwhile, the in vitro scratch wound healing assay demonstrated that small extracellular vesicles (sEVs) derived from human umbilical cord mesenchymal stem cells (hUCMSCs) significantly enhanced the migratory capacity of human dermal fibroblasts (HDFs), thereby accelerating wound closure. Treatment with both 75 µg/mL and 100 µg/mL concentrations of sEVs led to a marked reduction in wound area compared to the untreated control, with statistically significant differences observed by day 3. Notably, there was no significant difference between the two concentrations, suggesting that 75 µg/mL is sufficient to elicit an optimal therapeutic response within the evaluated timeframe. These findings underscore the robust regenerative potential of hUCMSC-sEVs in promoting fibroblast migration and cutaneous wound healing, reinforcing their promise as a therapeutic agent in skin tissue regeneration. Consistent with our findings, a previous study demonstrated that sEV treatment enhanced cellular migration toward the wound site, promoting partial gap closure compared to controls [72]. However, further investigations are needed to assess long-term cell viability and functional outcomes, which will be crucial for fully establishing the therapeutic potential of these scaffolds in wound healing.

## 5. Conclusions

This study successfully developed and characterized 3D bioprinted gelatin–GNP hydrogels incorporated with hUCMSC-sEVs, demonstrating their potential for wound healing applications in vitro. The optimized GNP-crosslinked gelatin hydrogel (GECL), particularly the 10% formulation, exhibited enhanced mechanical stability, controlled degradation, and favorable physicochemical properties such as swelling behavior, porosity, wettability, and WVTR—making it ideal for 3D bioprinting and future clinical use. The incorporation of hUCMSC-sEVs significantly improved the hydrogel’s regenerative potential by enhancing PHDF viability and attachment and accelerating wound healing in vitro, with 75 μg/mL identified as the optimal concentration. These results underscore the synergistic effect of a mechanically robust hydrogel combined with bioactive sEVs in supporting cell proliferation and tissue regeneration. Future work should evaluate the immunogenicity, safety, and mechanism of action of this hydrogel system through functional and in vivo studies. Investigating the controlled release profile of hUCMSC-sEVs under physiological conditions will further elucidate its therapeutic potential. Overall, the 10% GECL hydrogel functionalized with hUCMSC-sEVs offers a promising platform for advanced wound healing and personalized regenerative medicine.

## Figures and Tables

**Table 1:** Elemental analysis with EDX. All the hydrogels possessed elemental compositions including carbon (C), oxygen (O), nitrogen (N), sulfur (S), and sodium (Na).

Hydrogel	C (%)	O (%)	N (%)	S (%)	Na (%)
8% GNC	57.8 ± 0.4	25.2 ± 0.3	14.7 ± 0.5	1.5 ± 0.1	0.8 ± 0.1
8% GECL	54.5 ± 0.3	28.6 ± 0.3	15.6 ± 0.4	0.9 ± 0.1	0.4 ± 0.1
10% GNC	57.5 ± 0.3	29.2 ± 0.2	11.9 ± 0.3	1.0 ± 0.1	0.4 ± 0.0
10% GECL	51.6 ± 0.3	30.2 ± 0.3	17.1 ± 0.4	0.9 ± 0.1	0.3 ± 0.1

## Data Availability

The data will be available upon request.

## Abbreviations

The following abbreviations are used in this manuscript:

MSCs	Mesenchymal stem cells
sEVs	Small extracellular vesicles
hUCMSCs	Human umbilical cord mesenchymal stem cells
MSCs-sEVs	Mesenchymal stem cell-derived small extracellular vesicles
GE	Gelatin
GNP	Genipin
GECL	Gelatin crosslinking genipin
GNC	Gelatin non-crosslinking genipin
PHDFs	Primary human dermal fibroblasts
PBS	Phosphate-buffered saline
MTT	3-(4,5-Dimethylthiazol-2-yl)-2,5-Diphenyltetrazolium Bromide
M	Mean
mL	Milliliter
mm	Millimeter
nm	Nanometer
kDa	Kilodalton
SD	Standard deviation
*w*/*v*	Weight/Volume
P(n)	Cell Passage Number (n)
